# Challenges of Caregivers of Patients With Alzheimer’s Disease

**DOI:** 10.1093/geroni/igaa057.486

**Published:** 2020-12-16

**Authors:** Zara Sajwani

**Affiliations:** The University of Texas at Arlington, Allen, Texas, United States

## Abstract

The purpose of this presentation is to identify burden and problems experienced by caregivers of patients with Alzheimer’s Disease (AD). AD results in gradual deterioration of cognition, language, and memory that can impact an individual’s ability to independently perform daily functional activities (CDC, 2019). The role of caregivers is significant in providing assistance to the patients with chronic AD which can be a source of strain for caregiver population. About 16.3 million informal AD caregivers have spent 18.5 billion hours, which is equal to value of $234 billion, to assist patients with other dementia types and AD in 2018 (Alzheimer’s Association, 2019). In-depth literature synthesis was carried out using multiple databases. Recent and relevant articles were selected to be added in the review. Due to responsibility of constant vigilance of AD patients, the caregivers may overlook their self-care needs and detach themselves from social life. Literature analysis revealed common challenges and needs of AD care partners including limited social engagement, concerns of sexuality, and sleep problems. Understanding caregiver problems will help nurses and other health care professionals to support families by planning preventive measures. Resources can be invested to improve physical and mental well-being of caregivers. Researches can be planned to bridge the knowledge gap identified through literature review on this topic. References Alzheimer’s Association. (2019). Alzheimer’s disease caregivers. http://act.alz.org/site/DocServer/caregivers_fact_sheet.pdf?docID=3022 Centers for Disease Control and Prevention. (2019). Alzheimer’s disease and healthy aging. https://www.cdc.gov/aging/aginginfo/alzheimers.htm

